# Normal Exophthalmometry Values in Iranian Population: A Meta-analysis: A complete translation from Farsi

**DOI:** 10.18502/jovr.v16i3.9441

**Published:** 2021-07-29

**Authors:** Abbas Bagheri, Kourosh Shahraki, Amir Arabi, Mohsen Bahmani Kashkouli

**Affiliations:** ^1^Ophthalmic Research Center, Research Institute for Ophthalmology and Vision Science, Shahid Beheshti University of Medical Sciences, Tehran, Iran; ^2^Ophthalmic Plastic and Reconstructive Surgery Unit, Labbafinejad Eye Hospital, Shahid Beheshti University of Medical Sciences, Tehran, Iran; ^3^Ophthalmic Plastic and Reconstructive Surgery Unit, Rasoul-Akram Hospital, Iran University of Medical Sciences, Tehran, Iran

**Keywords:** Exophthalmometry, Hertel, Iran, Meta-analysis

## Abstract

There are limited studies on the normal values of eye protrusion in Iran. Systematic efforts to provide acceptable normal exophthalmometry values for Iranian population are required for a proper approach to orbital diseases.English and Farsi language publications in PubMed, the ISI Web of Knowledge database, Iranian SID, and Iran Medex were searched using the following keywords: “proptosis”, “eye protrusion”, “exophthalmous”, “Hertel exophthalmometer” and “Iran”. Four articles from 1995 to 2010 were found and included in the meta-analysis. Statistical analysis was performed using the Metan command within Stata 15.0 software.It included 3,696 subjects in whom the average eye protrusion was 16.5 mm (95% CI: 15.1–17.8) in men and 16.2 mm (95% CI: 14.6–17.7) in women (*P* = 0.5). Mean left and right eye protrusion were 16.3 (95% CI: 14.7–18.1) and 16.4 mm (95% CI: 14.8–17.7), (*P* = 0.3), respectively. While Iranian teenagers (13–19 years old) showed a mean value of 17.1 mm (95% CI: 15.0–19.1), older age group ( ≥ 20 years) showed a lower mean eye protrusion of 16.3 mm (95% CI: 14.8–17.7). Considering the two standard deviations, the highest normal value of eye protrusion in Iranian population is 20.1 mm.In conclusion,Iranian normal eye protrusion values were higher than Asians and lower than Caucasians.

##  INTRODUCTION

Different types of exophthalmometers can evaluate eye protrusion. These instruments all use facial bones as static points to estimate the distance between the corneal apex and the base.^[1]^ The reference points are the lateral orbital rims in the Hertel exophthalmometer, inferior and superior orbital rims in the Naugle exophthalmometer, and cheek and brow in the Mutch exophthalmometer.

Hertel exophthalmometer is the most commonly used exophthalmometer in clinical settings.^[4]^ It measures the distance between the apex of the cornea to the zygomatic arch on the lateral edge of the orbit. Although, previous studies have proved the reliability of the Hertel exophthalmometer,^[4,5]^ some have shown limited reproducibility among the examiners.^[6,7,8]^ Such a limitation usually roots from irregularity of the lateral orbital rims, parallax errors, compression of adjacent soft tissues, and the absence of a uniform measuring procedure.^[9]^

Eye protrusion varies in different populations and factors such as age, sex, and refractive error may affect it. The exophthalmometry value could be relative (right–left difference) or absolute (comparing with normal values of each population).^[10]^ Relative value of > 2 mm usually requires further investigations.^[6]^ Absolute exophthalmometry value is useful in the diagnosis of bilateral proptosis.^[10,11,12]^

There are limited studies on normal values of eye protrusion in Iran^[13,14,15,16]^ which were analyzed (meta-analysis) in order to define both normal relative and absolute eye protrusion values for Iranian teenagers and adults.

##  METHODS 

The present meta-analysis was performed following the preferred reporting items for meta-analyses checklist (PRISMA).^[17]^

### Search Strategies

The literature (prior to March 2019) was reviewed by searching PubMed, the ISI Web of Knowledge database, Iranian SID, and Iran Medex. The search strategy included the following keywords: “proptosis”, “eye protrusion”, “exophthalmos”, “Hertel exophthalmometer”, and “Iran”. Cross-references of any selected article were also used in the review. No language restriction was applied. Studies providing evidence-based information about standard values of Hertel exophthalmometry in Iranian population were selected. Of the 27 articles found through the search, 4 were included in this analysis. The first study was performed in 1995 and the last in 2010. We only included articles that had studied normal population in different cities of Iran.

### Data Extraction

Two of the authors reviewed the extracted data independently using a purpose-designed form. The following information were collected: first author, year of publication, geographic location, mean age, sample size, gender, different age groups, mean eye protrusion in right eye, mean eye protrusion in left eye, average eye protrusion in both eyes, and normal upper limit of eye protrusion. Data on eye protrusion were obtained using two-mirrored Hertel exophthalmometry in all studies. Eye protrusion is ethnic dependent; however, we did not study different Iranian ethnic groups separately.

### Statistical Analysis

Summary estimates of the pooled differences and mean protrusion of Iranian eyes for the normal value of Hertel exophthalmometry and related upper normal limits were combined using the inverse variance method.

Statistical meta-analysis was performed using the Metan command within Stata 15.0 software (StataCorp. 2017. Stata Statistical Software: Release 15. College Station, TX: StataCorp LLC). Between-study heterogeneity was assessed using Cochran Q and the inconsistency index (I ${}^{2}$ ). It was considered statistically significant when *p*-value < 0.05 or I ${}^{2}$ < 50%.^[18]^ Since a significant heterogeneity was found (I ${}^{2}$ > 50%), a random effect model was used to assess mean estimation and difference (MD), and 95% confidence intervals (95% CI). To explore possible foundations of heterogeneity, subgroup analyses were conducted for different age groups and genders, if applicable. To assess the influence of separate studies on the pooled measures, sensitivity analyses were directed by successively excluding studies. To further discover sources of heterogeneity, meta-regression analyses were performed. The potential publication bias was studied using the adjusted rank correlation test and the regression asymmetry test, respectively.^[18,19]^ In addition, we demonstrated the findings in each study as well as the pooled estimation in a forest plot. All statistical tests were two-tailed and *p* < 0.05 was considered statistically significant.

##  RESULTS

After a thorough text review, four articles published between 1995 and 2010 were included in the meta-analysis.

Bagheri et al's study^[13]^ included 926 randomly selected healthy individuals (481 men and 445 women) from Kashan Golabchi Clinic whose age ranged from 15 to 60 years. Those with ocular infections, history of orbital trauma, strabismus, endocrine system disease, significant myopia ( > 5 diopters [D]), children, and pregnant women were excluded from the study. One examiner using Hertel exophthalmometer in the sitting position performed all measurements. Each patient was examined once. Selected age groups in the study were 15–24 (*n* = 250), 25–34 (*n* = 346), 35–44 (*n* = 211), 45–54 (*n* = 91), and 55–64 (*n* = 28) years.

Hadaegh et al^[14]^ selected 404 normal subjects (15–75 years) by randomized stratified sampling from the East of Tehran who participated in Tehran Lipid and Glucose Study (TLG). Individuals with a history of ocular trauma, ocular surgery, thyroid disease, and those with > 7D myopia were excluded. Age groups were the same as the previous study and proptosis measurement was performed using Hertel exophthalmometer.

Tohidi et al^[15]^ recruited 1,303 healthy people from Bushehr (661 men and 642 women) with similar age groups and technique of Hertel exophthalmometery. Those with orbital disease and myopia of > 2D were excluded.

Kashkouli et al^[16]^ used stratified random sampling and recruited 1,603 normal subjects at different age groups from the West of Tehran.^[16]^ Three main age groups were determined according to the population poll statistics, including children (27.2%), teenagers (30%), and adolescents (42.8%). Average of two measurements done by an oculofacial plastic surgeon was recorded for each person. Exclusion criteria were similar to the previous studies.

Adding all the data of prior four studies resulted in 3,696 normal subjects (1,920 males and 1,776 females) of whom 2,774 were over the age of 20, 633 between 13 and 19, and 289 between 6 and 12 years [Table 1]. Subjects were from three cities in Iran (Tehran, Kashan, Bushehr).

**Table 1:** Normal eye protrusion values from the included studies*


**Study/age groups (yr)**	**Bagheri et al^[13]^ (2007)**				**Hadaegh et al^[14]^ (2002)**			**Tohidi et al^[15]^ (2013)**		
	**Kashan**				**East Tehran**			**Bushehr**		
	**Male**		**Female**		**Both genders**			**Both genders**		
	**OD**	**OS**	**OD**	**OS**	**OD**	**OS**	**Difference**	**OD**	**OS**	**Difference**
15–24	17.18 ± 1.99	17.15 ± 2.01	16.52 ± 1.62	16.59 ± 1.66	16.50 ± 4.2	16.8 ± 4.4	0.31 ± 1.5	19.51 ± 0.88	19.75 ± 0.85	0.24 ± 0.28
25–34	17.03 ± 2.15	17.01 ± 2.18	16.52 ± 1.70	16.58 ± 1.68	16.2 ± 4	16.5 ± 4.4	0.22 ± 1.6	18.8 ± 1.36	18.97 ± 1.38	0.16 ± 0.27
35–44	17.13 ± 2.2	17.17 ± 2.19	15.60 ± 3.97	15.67 ± 1.96	16 ± 4	16.1 ± 4.2	0.13 ± 1.6	17.93 ± 1.03	18.09 ± 1.02	0.15 ± 0.3
45–54	16.22 ± 2.38	16.22 ± 2.38	15.37 ± 2.40	15.34 ± 1.98	15.8 ± 4.8	16 ± 4.9	0.18 ± 1.4	17.27 ± 1.21	17.47 ± 126	0.2 ± 0.3
55–64	16.35 ± 2.62	15.50 ± 2.47	15.92 ± 2.12	15.58 ± 2.41	15.2 ± 4.4	15.5 ± 4.6	0.28 ± 1.6	16.27 ± 1.11	16.31 ± 1.16	0.04 ± 0.4
65–75	–	–	–	–	14.8 ± 2.5	15 ± 4.3	0.21 ± 1.8	15.98 ± 1.38	16.19 ± 1.61	0.2 ± 0.37
Total	16.99 ± 2.18	16.99 ± 2.18	16.10 ± 1.86	16.25 ± 1.84	16 ± 4.4	16.2 ± 4.5	0.23 ± 1.5	17.91 ± 1.55	18.08 ± 1.59	0.16 ± 0.31
*The values of one of the studies “Kashkouli et al^[16]^” was not provided in this table due to different age groups.				

**Table 2:** Eye protrusion values in different subgroups


**Group**	**Mean exophthalmometric values**	**Lower and upper limit of normal exophthalmometric values**
**Male**	16.5	12.55–20.45
**Female**	16.26	12.7–19.82
**Age 13–19 yr**	17.1	13.25–20.95
**Age > 20 yr**	16.32	12.62–20.02
**OD**	16.33	12.59–20.07
**OS**	16.44	12.64–20.24
**Total**	16.38	12.61–20.15

**Table 3:** Comparison of normal eye protrusion values in different ethnic groups


**Study**	**Geographic area**	**No.**	**Exophthalmometric value (Mean ± SD)**	**Age difference (mm)**	**Sex difference (mm)**	**Laterality difference (mm)**
**Present study**	Iran	3,696	16.38	13–19: 17.1	M: 16.5	OD: 16.33
		>20: 16.32	F: 16.26	OS: 16.44
**Erb et al^[12]^**	Asians in USA	89	14.4 ± 2.5	M: 15.5 ± 2.1	
			F: 13.5 ± 2.4	
**Chan et al^[20]^**	Sri Lanka	1,341	15.82 ± 2.73	M: 16.66	
			F: 15.27	
**Sarinnapakorn^[21]^**	Taiwan	277		M: 11.84	
			F: 11.44	
**Wu et al^[22]^**	Chinese Han	2,010	8–14: 13.7 ± 1.6	OD: 15 ± 1.5
		15–19: 15.1 ± 1.6	OS: 15 ± 2
		20–69: 15.7 ± 1.8	
		70–87: 15.3 ± 2.2	
**Bilen et al^[23]^**	Turkish	480		M: 13.49 ± 2.6	
			F: 13.44 ± 2.6	
**Dunsky et al^[4]^**	American Black	309		M: 18.20 ± 2.97	
			F: 17.46 ± 2.64	
**Migliori et al^[1]^**	American	681	In White: 16.5	White F: 14.4	
		In Black: 18.5	Black F: 17.8	
			OD: 15.27 ± 2.5
**Ibraheem et al^[24]^**	Nigeria	1,020		OS: 15.31 ± 2.4
**Mourits et al^[25]^**	Netherland	160		Upper limit, M: 20	
			Upper limit, F: 16	
**Jarusaitiene et al^[26]^**	Lithuania	397	14.91 ± 1.68		
			M: 15.18 ± 2.16	
**Bolaños et al^[27]^**	Mexico	301		F: 14.82 ± 1.98	
*M, male; F, female; OD, right eye; OS, left eye						

**Figure 1:**
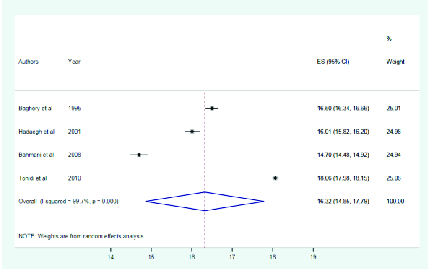
Mean eye protrusion in adults > 20 year old.

**Figure 2:**
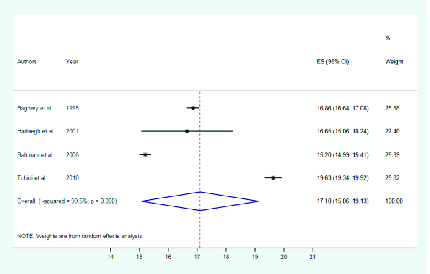
Mean eye protrusion in 13–19 years age group.

**Figure 3:**
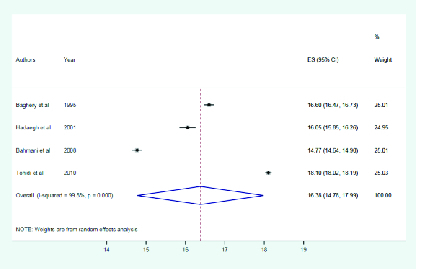
Mean eye protrusion in total population.

Pooled estimates of the primary subgroup analyses showed that the mean eye protrusion of Iranian adults ( ≥ 20 years of age) and teenagers (13–19 years) were 16.3 (95% CI: 14.85–17.79) and 17.1 mm (95% CI: 15.06–19.13), respectively [Figures 1 and 2]. However, the mean eye protrusion difference between the two eyes was not significant (*P* = 0.2). The mean eye protrusion of the right and left eye were 16.3 (95% CI: 14.70–18.18) and 16.4 mm (95% CI: 14.86–17.79), respectively. The mean exophthalmometric value in Iranian population was 16.3 mm (95% CI: 14.78–17.99) [Figure 3]. It was 16.5 mm (95% CI: 15.11–17.89) in men and 16.2 mm (95% CI: 14.65–17.72) in women. Gender differences are demonstrated in Table 2.

##  DISCUSSION

Iranian normal eye protrusion values were higher than that of Asians and lower than that of Caucasians [Table 3]. Results of this meta-analysis on 3,696 normal Iranians showed that the mean eye protrusion was neither significantly different between men (16.5 mm) and women (16.2 mm) nor between the right (16.3) and the left (16.4 mm) eyes. The mean eye protrusion value decreased from 17.1 mm in teenagers (13–19 years) to 16.2 mm in adults ( ≥ 20 years).

Kashkouli et al^[16]^ reported the normal eye protrusion in Iranian children (6–12 years) as 14.1 ± 1.8 mm which was less than that found in the Chinese population (14.4 mm), Arabian (15.4 mm), American children aged 5–8 years old (14.4 mm), and American children aged 9–12 years (15.2 mm), but it was more than the Indian (13 mm), Italian (between 9.1 and 11.7 mm), and White European (14 mm) children. Mean Hertel exophthalmometry value increased in the second decade of life (13–19 years) in Iranian population.

In a study by Erb and colleagues in Asian normal subjects,^[12]^ the mean eye protrusion calculated with Hertel exophthalmometer was 14.4 ± 2.5 mm, which is about 2 mm less than the normal Iranian population. Contrary to our study, significant statistical differences were observed between men (15.5 ± 2.1) and women (13.5 ± 2.4 mm) in Erb's study. In the same study, there was no more than 1-mm difference between the two eyes in all the cases.

In addition, the upper limit of normal exophthalmometric values in Asian normal adults was 19.4 mm, which was slightly higher in men than in women (19.19 vs 18.3 mm). It is about 2 mm less than the normal Caucasian population. This value was 20.15 mm in Iranian population.In a study by Chan et al^[20]^ including 1,341 adult cases from Sri Lankan population, the mean exophthalmometric value was 15.82 ± 2.73 mm, which was higher in men than in women. This difference was not significant between different Sri Lankan breeds (Sinhalese, Tamils, and Moors) but it had a significant association with age, sex, height, weight, body mass index (BMI), and axial length. According to the findings of our study, the normal amount of eye protrusion is higher in males than in females, although these values are greater than the Sri Lankan race.

In Sarinnapakorn's study in Taiwan,^[21]^ the normal protrusion of the eyes was 11.84 mm in men and 11.44 mm in women, which is about 5 mm less than the Iranian population.

In a study by Wu et al in 2010 in Han,^[22]^ Chinese native population in northeastern China had a mean of 15 ± 1.5 and 15 ± 2 mm protrusion in the left and right eyes, respectively, and the upper limit for the left and right eyes was 18.8 and 19 mm. Gender had no effect on the protrusion of the eyes but age had a significant statistical relationship with it. These values are similar to those of other studies in East Asia, and less than those of normal Iranian population.

In the study of Bilen et al in northern Turkey,^[23]^ mean values of eye protrusion were 13.49 ± 2.6 and 13.44 ± 2.6 in men and women, respectively, which showed no significant difference between the two genders. The results of their study are similar to those of Beden's study in Turkey. Overall, Turkish values are about 3 mm less than the Iranian population.

In Dunsky's study performed on American normal Black adults,^[4]^ the mean eye protrusion was 18.2 mm in men and 18.46 mm in women and the upper limits in men and women were 24.44 and 22.74 mm, respectively, which are higher than those in Iranian population with an average of 3.5 mm.Migliori and Gladstone studied 681 American adults (327 White and 354 Black)^[1]^ and reported a mean of 16.5 and 18.5 mm for normal eye protrusion in White and Black men, respectively. The value was 14.4 for White women and 17.8 for Black women, while abnormal values for White men, Black men, White women and Black women were 21.7, 24.7, 20.1, and 23 mm respectively. Accordingly, a similarity was observed between American White population and Iranian population.

In Ibraheem's study,^[24]^ 1,020 normal subjects in Nigeria had an average values of 15.27 ± 2.5 and 15.31 ± 2.4 mm in the right and left eyes, respectively, which were lower than reported values from African Americans, Chinese, Caucasian, and Iranian population.In a study by Mourits et al on normal population of Netherlands,^[25]^ the upper normal limit for exophthalmometry with Hertel was reported as 16 mm in women and 20 mm in men. The values in our study were 19.8 mm for women and 20.4 mm for men, which show that in Iranian population, unlike the Dutch population, there is no significant difference in eye protrusion between men and women.

Jarusaitiene et al, in their study on normal population in Lithuania,^[26]^ reported a mean eye protrusion was 14.91 ± 1.68 mm. Although eye protrusion was higher in women than in men, and in the right eye in comparison with the left eye, the difference was not statistically significant. In this study, there was a significant association between the range of protrusion and the age, weight, and height. In the Iranian population, in contrast to the study of Lithuania, the mean eye protrusion was higher in men than in women, and was greater in the left eye compared to the right; however, these values were not statistically significant.

Bolaños and colleagues reported mean eye protrusion in the normal population of Mexico; 15.18 mm in men and 14.38 mm in women,^[27]^ which are lower than those of normal Iranian population.

Differences in baseline features between individuals were partially adjusted because of different study populations and formats.

In conclusion, this meta-analysis provides the literature with cut-off values for normal eye protrusion in Iranian population.

##  SUMMARY

There are limited studies on normalvalues of eye protrusion in Iran, which were analyzed (metaanalysis) in order to define both normal relative and absolute eye protrusion values for Iranian teenagers and adults. Systematic efforts to provide acceptable normal exophthalmometry values for Iranian population are required for a proper approach to orbital diseases.

Iranian normal eye protrusion values were higher than that of Asians and lower than that of Caucasians.

##  Financial Support and Sponsorship

Nil.

##  Conflicts of Interest

There are no conflicts of interest.

## References

[B1] Migliori ME, Gladstone GJ (1984). Determination of the normal range of exophthalmometric values for
black and white adults. Am J Ophthalmol.

[B2] Cole HP 3rd, Couvillion JT, Fink AJ, Haik BG, Kastl PR (1997). Exophthalmometry: a comparative study of the Naugle and Hertel
instruments. Ophthalmic Plast Reconstr Surg.

[B3] Naugle TC Jr, Couvillion JT (1992). A superior and inferior orbital rim-based exophthalmometer
(orbitometer). Ophthalmic Surg.

[B4] Dunsky IL (1992). Normative data for hertelexophthalmometry in a normal adult black
population. Optom Vis Sci.

[B5] Ameri H, Fenton S (2004). Comparison of unilateral and simultaneous bilateral measurement
of the globe position, using the Hertelexophthalmometer. Ophthalmic Plast Reconstr Surg.

[B6] Kashkouli MB, Beigi B, Noorani MM, Nojoomi M (2003). Hertelexophthalmometry: reliability and interobserver
variation. Orbit.

[B7] Lam AK, Lam CF, Leung WK, Hung PK (2009). Intra-observer and inter-observer variation of
Hertelexophthalmometry. Ophthalmic Physiol Opt.

[B8] Musch DC, Frueh BR, Landis JR (1985). The reliability of hertelexophthalmometry. Observer variation between physician and lay readers
Ophthalmology.

[B9] Kim IT, Choi JB (2001). Normal range of exophthalmos values on orbit computerized
tomography in Koreans. Ophthalmologica.

[B10] Lee BJ (2012). Orbital Evaluation.

[B11] Sisler HAJF, Trokel SL, Duane’s clinical ophthalmology Volume 2.

[B12] Erb MH, Tran NH, McCulley TJ, Bose S (2003). Exophthalmometry measurements in Asians. Invest Ophthalmol Vis Sci.

[B13] Bagheri A, Behtash F (2007). Normal exophthalmometry range in Kashan city. J GUMS.

[B14] Farzad H, Fereydoun A, Farzad P, Maryam T (2003). Determination of the normal exophthalmometric values in Tehran
population/Iran. ISMJ.

[B15] Tohidi M, Hormozi MK, Nabipour I, Vahdat K, Assadi M, Karimin F, et al (2013). Determination of normal values for eye protrusion in Bushehr port
population. ISMJ.

[B16] Kashkouli MB, Nojomi M, Parvaresh MM, Sanjari MS, Modarres M, Noorani MM (2008). Normal values of hertelexophthalmometry in children, teenagers,
and adults from Tehran, Iran. Optom Vis Sci.

[B17] Moher D, Shamseer L, Clarke M, Ghersi D, Liberati A, Petticrew M, et al (2015). Preferred reporting items for systematic review and meta-analysis
protocols (PRISMA-P) 2015 statement. Syst Rev.

[B18] Li G, Li Y, Chen X, Sun H, Hou X, Shi J (2016). Circulating tocopherols and risk of coronary artery disease: a
systematic review and meta-analysis. Eur J Prev Cardiol.

[B19] Begg CB, Mazumdar M (1994). Operating characteristics of a rank correlation test for
publication bias. Biometrics.

[B20] Chan W, Madge SN, Senaratne T, Senanayake S, Edussuriya K, Selva D, et al (2009). Exophthalmometric values and their biometric correlates: The
Kandy Eye Study. Clin Exp Ophthalmol.

[B21] Sarinnapakorn V, Sridama V, Sunthornthepvarakul T (2007). Proptosis in normal Thai samples and thyroid
patients. J Med Assoc Thai.

[B22] Wu D, Liu X, Wu D, Di X, Guan H, Shan Z, et al (2015). Normal values of Hertel exophthalmometry in a Chinese Han
population from Shenyang, Northeast China. Sci Rep.

[B23] Bilen H, Gullulu G, Akcay G (2007). Exophthalmometric values in a normal Turkish population living in
the northeastern part of Turkey. Thyroid.

[B24] Ibraheem WA, Ibraheem AB, Bekibele CO (2014). Exophthalmometric value and palpebral fissure dimension in an
African population. Afr J Med Health Sci.

[B25] Mourits MP, Lombardo SH, van der Sluijs FA, Fenton S (2004). Reliability of exophthalmos measurement and the exophthalmometry
value distribution in a healthy Dutch population and in Graves’
patients. An exploratory study Orbit.

[B26] Jarusaitiene D, Lisicova J, Krucaite A, Jankauskiene J (2016). Exophthalmometry value distribution in healthy Lithuanian
children and adolescents. Saudi J Ophthalmol.

[B27] BolaÃ±os Gil de Montes F, PÃ©rez Resinas FM, RodrÃ­guez GarcÃ­a M, GonzÃ¡lez Ortiz M (1999). Exophthalmometry in Mexican adults. Rev Invest Clin.

